# Effects of Daily Low-Dose Date Consumption on Glycemic Control, Lipid Profile, and Quality of Life in Adults with Pre- and Type 2 Diabetes: A Randomized Controlled Trial

**DOI:** 10.3390/nu12010217

**Published:** 2020-01-15

**Authors:** Tariq A. Alalwan, Simone Perna, Qaher A. Mandeel, Aalaa Abdulhadi, Adel Salman Alsayyad, Giuseppe D’Antona, Massimo Negro, Antonella Riva, Giovanna Petrangolini, Pietro Allegrini, Mariangela Rondanelli

**Affiliations:** 1 Department of Biology, College of Science, University of Bahrain, P.O. Box 32038, Sakhir, Bahrain; simoneperna@hotmail.it (S.P.); mandeelq@gmail.com (Q.A.M.); ala2_ba7rain@hotmail.com (A.A.); 2 Public Health Directorate, Ministry of Health, Manama, Bahrain; aassayyad2010@gmail.com; 3 Department of Family and Community Medicine, College of Medicine and Medical Sciences, Arabian Gulf University, Manama, Bahrain; 4 CRIAMS-Sport Medicine Centre, University of Pavia, 27058 Voghera, Italy; gdantona@unipv.it (G.D.); massimo.negro@unipv.it (M.N.); 5 Research and Development Department, Indena SpA, 20139 Milan, Italy; Antonella.riva@indena.com (A.R.); Giovanna.petrangolini@indena.com (G.P.); Pietro.allegrini@indena.com (P.A.); 6 IRCCS Mondino Foundation, 27100 Pavia, Italy; mariangela.rondanelli@unipv.it; 7 Department of Public Health, Experimental and Forensic Medicine, Unit of Human and Clinical Nutrition, University of Pavia, 27100 Pavia, Italy

**Keywords:** dates, obesity, diabetes, inflammation, antioxidants

## Abstract

Dates have a low glycemic index and are a source of antioxidants but, nevertheless, contain more than 70% sugar. This study aims to assess the effects of date consumption (three dates daily) on glycemic profile (HbA1c), body mass index (BMI), quality of life, and lipid profile, including total cholesterol, triglyceride, high-density lipoprotein (HDL), and low-density lipoprotein (LDL) in terms of safety for type 2 diabetic mellitus (T2DM) subjects. A randomized controlled trial was conducted with a sample of 100 T2DM subjects (39 male and 61 female) randomly assigned in two groups. The first group received three dates daily for 16 weeks, and the control group avoided date consumption. After a 16-week follow-up period, the study results showed an improvement of lipid profile with a statistically significant decrease in total cholesterol of ∆ = −0.209 mmol/L (confidence interval (CI) 95% −0.358, −0.059; *p* < 0.05) and in LDL of ∆ = −0.171 mmol/L (CI 95% −0.358, 0.016) in the group receiving three dates daily. Intra-group mean differences of BMI were not statistically different in both groups after 16 weeks of date consumption. Even HbA1c did not change, both within and between groups after date consumption (∆ = 0.087%; CI 95% −0.086, 0.261). Between groups, mean difference changes (intervention minus control) showed a statistically significant improvement of quality of life index of ∆ = ± 30.66 points (CI 95% 12.45, 48.23) due to the consequent improvement in mental health. Although the definitive effect of dose/intake response of date consumption on Hb1Ac, lipid profile, and BMI in T2DM subjects is still to be established, the study suggests that dates could potentially have a beneficial effect on lipid profile, especially in reducing total cholesterol and elevating HDL, because of its high polyphenolic content. In addition, a low–moderate consumption of dates did not impact glucose levels because of dates’ low glycemic index.

## 1. Introduction

Date palm (*Phoenix dactylifera* L.) is one of the most important fruit crops in the Middle East and North Africa that produce edible and delicious dates. Date palms are spread across Iraq, Iran, Saudi Arabia, Egypt, Tunisia, Algeria, Libya, United Arab Emirates (UAE), Bahrain, and Oman. All date varieties have four distinct stages of maturity, namely, kimri (immature and inedible), khalal (partially ripe), rutab (soft and ripe), and tamar (ripe and dried) [1]. The date fruit is a traditional delicacy and a seasonal heritage food appreciated mainly for its nutritional and medicinal values, a fact that is well documented in the Holy Quran. The Islamic tradition of breaking a fast with dates during the holy month of Ramadan is observed in all Arab and Islamic countries [2].

Nutritional analysis showed that dates are high in carbohydrates and sugar. Dates consist of more than 70% sugar, consisting mainly of glucose, fructose, and a small amount of sucrose [3]. Dates are also rich in fibers, vitamins, and minerals. Dates contain many nutrients such as calcium, magnesium, amino acids, iron, zinc, potassium, phosphorous, and selenium [4]. Depending on the date variety, there are different levels and patterns of bioactive non-nutrient phytochemicals, including carotenoids, polyphenols, and particularly phenolic acids, along with flavonoids [5,6]. Four free phenolic acids (protocatechuic acid, vanillic acid, syringic acid, and ferulic acid) and nine bound phenolic acids (gallic acid, protocatechuic acid, *p*-hydroxybenzoic acid, vanillic acid, caffeic acid, syringic acid, *p*-coumaric acid, ferulic acid, and *o*-coumaric acid) were identified in Omani date varieties [4].

Recent studies have shown that these constituents of dates act as potent antioxidants, antiglycemics, and anti-inflammatories, providing suitable nutritional therapy for different diseases such as cancer, diabetes, and dyslipidemia [5,6,7,8]. Worldwide, an estimated 425 million adults aged 20–79 years have type 2 diabetes mellitus (T2MD) [9]. As such, there are concerns and expressed reservations in the medical community about the consumption of a certain amount of dates among T2DM patients [5]. To our knowledge, there are no available data showing the association between date consumption and T2DM, obesity, and lipid profile.

Dates, because of their high fiber and phenolic contents, could play a potent role in the prevention of chronic diseases such as cardiovascular disease (CVD), atherosclerosis, hypertension and T2DM. Prevention of CVD may be due to the inhibition of platelet aggregation as well as the oxidation of low-density lipoprotein (LDL). Phenolics, because of their anti-inflammatory and antithrombotic effects, may be able to reduce blood pressure [5,10]. Such effects are undoubtedly attributed to the antioxidant properties of phenolic compounds by chelating metal ions and scavenging free radicals, as previously reported in different varieties of dates [11,12,13].

Preliminary data in the literature suggest that date fruit supplementation may exert maximum serum cholesterol, triglyceride, and LDL reduction potential through several mechanisms that modulate cholesterol absorption and metabolism [14,15]. In addition to these animal studies, a recent clinical study with 60 geriatric patients having borderline high LDL (130 to 159 mg/dL) revealed significant reductions of LDL plasma levels after a daily intake of 20–35 g of dates for six months [16]. In addition to these benefits, polyphenolic compounds from dates are able to decrease the postprandial hyperglycemia in T2MD by inhibiting the carbohydrate-hydrolyzing enzymes α-glucosidase and α-amylase [17]. Date fruit extracts can also suppress hypersensitive immune responses because of their anti-inflammatory, antiallergic, and antimodulatory qualities [18]. Moreover, the study by Hussein et al. [19] also confirmed the anti-hyperglycemic effects of date fruit extracts on liver function in diabetic rats.

Phytochemicals present in dates could also be used as antiobesetic agents, as demonstrated by the inhibitory effect of pits from various date palm varieties against lipase activity [20]. The effectiveness of the date pit extract against lipase activity strongly suggests that dates through their phytochemical constituents could minimize the absorption of lipids and energy intake from food.

The aim of this research is to assess the effect of date consumption on glycemic profile (HbA1c), body mass index (BMI), quality of life, and lipid profile in Bahraini adult T2DM patients.

## 2. Materials and Methods

### 2.1. Study Design

This study was a randomized controlled trial. The protocol of the present study was approved by the Ministry of Health in Bahrain and the Ethics Committee of the Department of Biology at the University of Bahrain. The study was conducted in full compliance with the Declaration of Helsinki and Ethical Guidelines. The University Trial Registration Number is AURS/496/2018.

Participants were enrolled from January 2018 to September 2018, and the end of the trial was in January 2019. Written informed consent was obtained from all subjects participating in the study.

### 2.2. Inclusion and Exclusion Criteria

Subjects with the following criteria were eligible: diagnosed with T2DM according to the diabetes diagnostic criteria (current HbA1c >6.0% and <10.0%), aged between 20 and 65 years, BMI >22.0 kg/m^2^, and an estimated glomerular filtration rate >50.0 mL/min/1.73 m^2^.

Subjects with the following criteria were excluded: type 1 diabetes, history of diabetic ketoacidosis, diabetic coma or precoma within 6 months prior to the date of consent, serious infections, surgery, serious trauma requiring insulin therapy, moderate or higher renal dysfunction (male serum creatinine (Cre) level ≥1.3 mg/dL and female Cre ≥1.2 mg/dL), hemodialysis treatment (including peritoneal hemodialysis), severe liver injury, and history of serious vascular complications (stroke, myocardial infarction, and heart failure) requiring hospital admission.

### 2.3. Randomization

This study was a randomized, monocenter (University of Bahrain), not blinded, and controlled clinical trial. Assignment to the 3 dates daily group or the control group (no intake of dates) was according to a computer-generated randomization scheme (1:1) done by the nutritionist and statistician.

### 2.4. Procedures

T2DM subjects in the intervention group received 3 dates daily for 16 weeks, while those in the control group were initially advised to avoid date consumption.

The variety of dates used in the study was Khudary cultivar dates, which is widely consumed among Bahrainis. The same batches of packaged dates (tamar stage) purchased from the local market, imported from Al-Qassim, Saudi Arabia, were used for this investigation.

During the study period, diet and exercise therapy remained the same as before the study initiation.

### 2.5. Outcomes

The primary outcome was to assess the change in HbA1c levels in the intervention group that received 3 dates daily for 16 weeks. Secondary outcomes included changes in BMI values, body composition, total cholesterol, LDL cholesterol, fasting, triglycerides, HDL cholesterol, and quality of life Short Form 36 (SF-36) index. We assessed how safe our study was by recording all adverse events seen during the study.

### 2.6. Experimental Design and Diets

A two-arm, randomized control trial was performed for a period of 16 weeks. Three dates daily were provided as part of a breakfast meal. The subjects were instructed to maintain their regular dietary habits, except for breakfast, and to record and take pictures of the contents of daily meals, snacks, and beverages during the intervention period.

### 2.7. Study Visits and Blood Samples

Subjects attended two visits to the laboratory, spaced 16 weeks apart. Participants were not permitted to eat or drink (except for water) and to smoke 12 h prior to the blood sample being taken between 7:00 and 8:30 a.m. Height and body weight were measured in order to calculate the BMI.

Biochemical analyses on total cholesterol, HDL, and triglycerides were measured by commercially available enzymatic assays using an autoanalyzer. LDL was calculated using the formula of Friedewald [21]. HbA1c and fasting blood glucose were also recorded.

### 2.8. Health-Related Quality of Life

The participants were tested with the SF-36 health survey to assess their quality of life [22]. This questionnaire is a valid generic measure that is used for rating health-related quality of life in several research fields because of its validity, high internal consistency, and high test–retest reliability. In particular, it has been evaluated in the area of mental health as a single item. The SF-36 scales were summarized in two dimensions.

### 2.9. Dietary and Physical Activity Assessments

Basal dietary analysis was conducted a week before the test period. The diet diary was collected every weekday, and the daily intake of each component was calculated by an experienced dietitian on the basis of the food composition tables for the Bahrain [23]. The level of physical activity of the volunteers was sedentary (physical activity level (PAL) 1.40–1.69) at baseline.

Volunteers were advised to continue current levels of physical activity during the clinical trial. Furthermore, physical activity was monitored on a weekly basis with self-assessment done by the International Physical Activity Questionnaire (IPAQ) [24].

### 2.10. Sample Size

The sample size was determined based on the previous study by Verma et al. [16]. Based on this, the sample size was calculated as 100 subjects (50 subjects in each group by using a two-sided two-sample *t*-test at 0.05 level of significance, taking into consideration a dropout rate of 10%, and 90% power).

### 2.11. Statistical Analysis

The statistical analysis and reporting of this study were conducted in accordance with the consolidated standards of reporting trials (CONSORT) guidelines [25], with the primary analysis based on the full analysis set. For the baseline variables, summary statistics employed frequencies and proportions for categorical data and mean and standard deviation (SD) for continuous variables. Baseline variables were compared using chi-square or Fisher’s exact tests for categorical outcomes and unpaired *t*-tests for continuous variables, as appropriate. In the primary analysis, the baseline-adjusted means and 95% confidence interval (CI) estimated by analysis of covariance (ANCOVA) with the change in the primary outcome at 16 weeks were compared between the 3 dates daily group and control group. The comparisons were adjusted for age and gender, ANCOVA was also used for the secondary outcomes at each time point (16 weeks). The adverse events analysis and safety analysis were performed by Fisher’s exact test. All *p* values were two-sided. A *p* value <0.05 was considered statistically significant. All analyses were performed by SPSS 21 software (IBM, Chicago, USA).

## 3. Results

**Figure 1:**
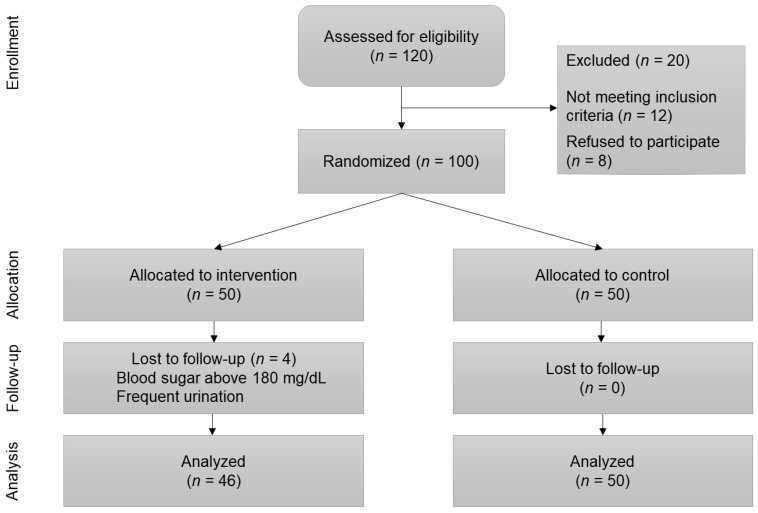
Flow chart of the study.

As shown in Figure 1, 12 subjects were excluded from the analysis due to non-adherence to the protocol. After exclusion, the remaining 100 subjects were randomly allocated into the date-consumers group (*n* = 50) and the control group (*n* = 50). Only 46 subjects in the intervention group were analyzed, as four patients were lost in the follow-up, while all subjects in the control group completed the follow-up.

The baseline parameters of the study are displaced in Table 1. There was not any statistically significant difference between the two groups at baseline for each outcome. Among the 39 male and 61 female subjects included in the analysis (most of whom were sedentary), the mean age was 55 years, with a SD of 3.35 years, and a mean BMI of 29.22 ± 6.11 kg/m^2^.

### 3.1. HbA1c and Serum Lipid Profiles

As shown in Table 2, the level of HbA1c remained stable in the group consuming dates, with ∆ = 0.043% (−0.082, 0.169) and *p* = 0.311, while with the control group, ∆ = −0.044% (−0.164, 0.076) and *p* = 0.554. Between-group analysis showed a slight reduction (but not statistically significant) of ∆ = –0.087% (−0.086, 0.261; *p* = 0.296) between the groups.

After a follow-up period of 16 weeks, the findings showed an improvement of lipid profile with a statistically significant decrease in total cholesterol in the group receiving 3 dates daily (∆ = −0.209 mmol/L (−0.358, −0.059), *p* < 0.05; versus nondates consumers that found ∆ = 0.024 mmol/L (−0.167, 0.119), *p* = 0.115) and a suggestive, but not statistically significant decrease in LDL in the intervention group of ∆ = −0.171 mmol/L (−0.358, 0.016). No effects on other biomarkers tested, such as on glycemia, were detected either within groups or between groups.

### 3.2. Quality of Life

Table 2 shows a statistically significant increase of quality of life index (intervention minus control): ∆ change mean differences equal to 30.66 points (12.45, 48.23). Regarding the SF-36, the between-groups change was 5.64 (0.57, 10.70) points.

### 3.3. Safety

No relevant side effects were observed in the intervention group.

### 3.4. Monitoring the Physical Activity

IPAQ score of the overall groups showed a non-statistically significant difference in physical activity for each weekly follow-up. The mean level score of physical activity intragroup (time × interventions) did not statistically differ significantly (intervention: *p* = 0.405; control: *p* = 0.302).

### 3.5. Dietary Assessment

Changes in nutritional intake in the intervention and control groups did not differ between groups either. The dietary intake of both groups (not including the intervention or control) is shown in Supplementary Table S1.

**Figure 2:**
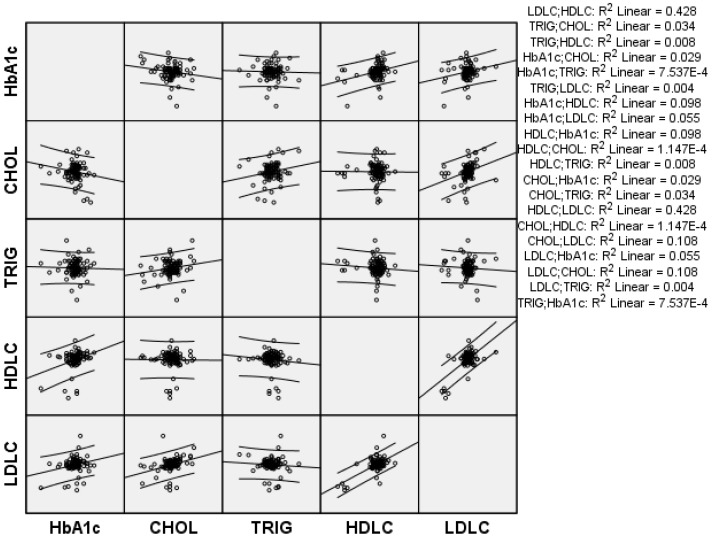
Plot matrix of correlation between ∆ change mean differences in the intervention group of lipid and glycemic profiles.

Figure 2 describes the correlation among the mean differences of HbA1c, total cholesterol, HDL, LDL, and triglycerides (R^2^) for the intervention group before and after the intervention. R^2^ is computed as the partial correlation, adjusted for gender. No statically significant associations were detected according to the level of R^2^.

## 4. Discussion

The present study shows for the first time in literature that a daily low intake of dates in T2DM subjects did not affect an increase in HbAlc levels, and there was no evidence to discourage individuals with diabetes from avoiding dates in moderation. Furthermore, the daily low intake of dates did not affect the BMI, and it seems promising in the enhancement of the lipid profile.

The study findings revealed that date consumption in T2DM subjects was associated with a statistically significant decrease in total cholesterol and a suggestive (but not significant) decrease in LDL after the consumption of three dates per day for 16 weeks in the intervention group. This was probably because dates are a good source of dietary fiber, which has been shown to be hypocholesterolemic in animals [26]. A similar effect was reported during 13 weeks of dates supplementation in hamsters [14]. Another possible explanation could be that the increased concentrations of plasma carotenoids released during the digestion of dates competed with dietary lipids for assimilation and transport in lipoproteins, subsequently leading to lower cholesterol levels [27]. Contrary to our results, the study of Rock et al. [28] found no statistically significant effect of date consumption in healthy human subjects on total cholesterol and HDL level. Nonetheless, the same study showed a significant decrease in triglycerides following the consumption of dates [28].

Further, we found that date consumption for 16 weeks had no effect on body mass index in both groups either before or after date consumption. This could be attributed to the high polyphenol content present in dates. Similarly, Rock et al. [28] showed that date consumption for four weeks did not significantly affect the BMI of healthy participants.

Moreover, the BMI of healthy subjects consuming approximately 70 g dates daily was not accompanied by any weight change at the end of the three-week study period [29]. Interestingly, Al-Mssallem et al. [30] also concluded that the association between date consumption and weight gain was very weak. The authors further indicated that the consumption of dates was not responsible for weight gain among Saudi men and women [30].

In addition, this study showed that the consumption of dates boosted various measures of function and quality of life, particularly mental health as assessed by SF-36. Indeed, our study confirmed that the overall quality of life of subjects supplemented with three dates daily improved significantly. At the same time, the lack of date consumption in the control group affected the quality of life index of T2DM subjects. The probable explanation of this finding is that utilization of dates has cultural and religious functions among Muslims. The fruit is mentioned more than 20 times in the Quran, and it is usual to break the daily fast with dates during Ramadan. Moreover, it has a special social status among Bahrainis and Arabs in general, as dates and date-based foods are served during every auspicious occasion and event, such as weddings, births, family gatherings, and religious holidays [31].

In the present study, the consumption of dates showed no statistically significant increase or decrease of levels of HbA1c, LDL, and triglycerides between date consumers and nondate consumers, either before or after the intervention. This goes in line with the fact that fructose is less rapidly absorbed than glucose, which results in a relatively low glycemic index (GI) [32]. In addition, several clinical studies have reported that date consumption did not cause significant postprandial glucose excursions [32,33,34]. Furthermore, Al-Mssallemet et al. [35] recently provided evidence to conclude that date consumption had no association with the prevalence of T2MD in neighboring Saudi Arabia.

Limitations of the study were that the amount of dates consumed were limited to only three dates, which is on the low side, considering that, traditionally, large amounts of dates are consumed because of their religious and nutritional significance. In addition, we used only one variety of dates at the tamer stage. Last but not least, the overall macronutrient distribution and quantity of each group’s diet was not evaluated specifically for quality of fats, carbohydrates, and proteins (and relative foods). Consequently, conclusions from this study may not be applicable to all types (fresh and sun-dried) and varieties of dates.

## 5. Conclusions

The daily low intake of dates in T2DM subjects did not increase HbAlc levels, and there is no evidence to support discouraging people with diabetes from consuming dates in moderation. Our results showed clear benefits of date consumption on the lipid profile of diabetic individuals, particularly a significant reduction in total cholesterol and an increase in HDL cholesterol and quality of life. Furthermore, we have shown that date consumption does not result in increased triglyceride and LDL levels, or BMI. However, more long-term randomized clinically controlled intervention trials are needed to evaluate the effects of date consumption on serum glucose and insulin levels in T2DM individuals.

## Figures and Tables

**Table 1:** Clinical characteristics of the sample.

Variable	Control (*n* = 50)	Intervention (*n* = 50)	Total Sample (*n* = 100, Female = 61, Male = 39)	*p*-Value between Groups
Age (years)	56.86 ± 4.41	55.25 ± 2.71	56.00 ± 3.35	0.404
Total cholesterol (mmol/L)	3.68 ± 0.95	4.127 ± 1.04	3.39 ± 0.74	0.111
Triglyceride (mmol/L)	1.48 ± 0.57	1.693 ± 0.88	1.60 ± 0.78	0.125
HbA1c (%)	6.62 ± 0.67	6.59 ± 0.81	6.61 ± 74	0.584
HDL (mmol/L)	1.21 ± 0.29	1.149 ± 0.30	1.20 ± 0.36	0.794
LDL (mmol/L)	2.36 ± 0.70	2.3035 ± 0.92	2.30 ± 0.78	0.443
BMI (kg/m^2^)	29.92 ± 4.11	28.45 ± 7.69	29.22 ± 6.11	0.244
Total SF-36 (score)	284.89 ± 126.72	349.16 ± 146.71	315.47 ± 138.76	0.139
SF-36 mental health	48.00 ± 17.90	54.00 ± 16.20	51.00 ± 17.20	0.211
Glycemia (mg/dL)	109.44 ± 14.79	104.71 ± 13.16	107.38 ± 13.85	0.511

Abbreviations: BMI, body mass index; HbA1c, glycosylated hemoglobin; HDL, high-density lipoprotein; LDL, low-density lipoprotein; SF-36, Short Form 36.

**Table 2:** Within-group mean changes from baseline (from day zero to the end of the intervention) for clinical markers.

Variable	Control Intra-Group ∆ Change (SD)	Intervention Intra-Group Δ Change (SD)	Intervention Effect between Groups *p*-Value
HbA1c (%)	−0.044 (−0.164; 0.076)	0.043 (−0.082; 0.169)	0.087 (−0.086; 0.261)
Total cholesterol (mmol/L)	−0.024 (−0.167; 0.119)	**−0.209 (−0.358; −0.059)**	−0.185 (−0.392; 0.022)
Triglyceride (mmol/L)	−0.006 (−0.102; 0.090)	0.004 (−0.096; 0.105)	0.010 (−0.128; 0.149)
HDL (mmol/L)	0.032 (−0.060; 0.123)	−0.077 (−0.173; 0.018)	−0.109 (−0.241; 0.023)
LDL (mmol/L)	0.055 (−0.125; 0.235)	−0.171 (−0.358; 0.016)	−0.226 (−0.485; 0.034)
Total SF-36 (score)	−2.23 (−44.45; 47.29)	**28.34 (17.45; 37.59)**	**30.66 (12.45; 48.23)**
SF-36 mental health	−0.04 (−3.83; 6.75)	**5.60 (1.24; 7.96)**	**5.64 (0.57; 10.70)**
Glycemia (mg/dL)	6.71 (−2.51; 15.94)	−7.06 (−17.70; 3.57)	−13.77 (−28.73; 1.18)
BMI (kg/m^2^)	−0.50 (−2.00; 1.01)	0.53 (−0.25; 0.81)	−0.03 (−0.39; 0.33)

In bold: value with *p* < 0.05. Abbreviations: HbA1c, glycosylated hemoglobin; HDL, high-density lipoprotein; LDL, low-density lipoprotein; SF-36, Short Form 36.

## Supplementary Materials

The following is available online at https://www.mdpi.com/2072-6643/12/1/217/s1, Table S1: Daily dietary intakes of subjects at baseline and during the 16-week intervention.
