# Supplementary material for: Effects of Daily Low-Dose Date Consumption on Glycemic Control, Lipid Profile, and Quality of Life in Adults with Pre- and Type 2 Diabetes: A Randomized Controlled Trial
Source: Nutrients. 2020 Jan 15;12(1):217. doi: 10.3390/nu12010217 (PMC7019638; doi:10.3390/nu12010217)
Supplement: Supplementary file 1 [file nutrients-12-00217-s001.pdf]

**Table S1.** Daily dietary intakes of subjects at baseline and during the 16-week intervention.

| | | BASAL (control) | 16 week(control) | BASAL(3 dates/day) | 16 week(3 dates/day) |
|---|---|---|---|---|---|
| Energy | kcal | 2216±55.44 | 2.230±64.63 | 2.233±61.00 | 2.230±64.63 |
| Protein | g | 81.18±12.70 | 87.86±17.43 | 86.86±16.43 | 89.86±18.43 |
| Fat | g | 70.86±15.40 | 76.36±15 | 72.46±17 | 70.36±12 |
| Saturated fatty acids | g | 22.56±4.70 | 23.43±6.30 | 29.43±6.65 | 21.43±8.30 |
| Cholesterol | mg | 379.61±20.44 | 361.54±45.55 | 371.54±67.88 | 361.54±55.77 |
| Carbohydrate | g | 356.86±45.44 | 292.86±71 | 272.86±67 | 262.86±43 |
| Total dietary fibers | g | 14.33±4.90 | 16.66±3.06 | 14.56±3.06 | 11.60±3.06 |

*Data compared to basal by Wilcoxon signed rank test. No statistically differences were found pre-after
